# Plasma protein C levels in immunocompromised septic patients are significantly lower than immunocompetent septic patients: a prospective cohort study

**DOI:** 10.1186/1756-8722-2-43

**Published:** 2009-10-19

**Authors:** Rakshit Panwar, Bala Venkatesh, Peter Kruger, Robert Bird, Devinder Gill, Leo Nunnink, Goce Dimeski

**Affiliations:** 1 Intensive Care, Monash Medical Centre, Clayton, VIC 3168, Australia; 2 Intensive Care, Princess Alexandra Hospital Princess Alexandra Hospital, Brisbane, Australia; 3 Intensive Care, Wesley Hospital, Brisbane, Australia; 4 Department of Intensive Care, University of Queensland, Brisbane, Australia; 5 Department of Hematology, Princess Alexandra Hospital, Brisbane, Australia; 6 Department of Chemical Pathology, Princess Alexandra Hospital, Brisbane, Australia

## Abstract

**Introduction:**

Activated Protein C [APC] improves outcome in immunocompetent patients with severe sepsis particularly in those who are perceived to have high mortality risk. Before embarking on a trial of APC administration in immunocompromised septic patients, a preliminary study on plasma levels of protein C in this cohort is essential.

**Objective:**

To assess serum Protein C concentrations in immunocompromised patients as compared to immunocompetent patients during sepsis, severe sepsis, septic shock and recovery.

**Methods:**

Prospective cohort study in a tertiary hospital. Patients satisfying inclusion criteria were enrolled after informed consent. Clinical variables were noted with sample collection when patients met criteria for sepsis, severe sepsis, septic shock and recovery. Protein C levels were measured using monoclonal antibody based fluorescence immunoassay.

**Results:**

Thirty one patients participated in this study (22 immunocompromised, 9 immunocompetent). Protein C levels were found to be significantly lower in the immunocompromised group compared to the immunocompetent group, particularly observed in severe sepsis [2.27 (95% *CI: *1.63-2.9) vs 4.19 (95% *CI: *2.87-5.52) mcg/ml] (p = 0.01) and sepsis [2.59 (95% *CI: *1.98-3.21) vs 3.64 (95% *CI: *2.83-4.45) mcg/ml] (p = 0.03). SOFA scores were similar in both the groups across sepsis, severe sepsis and septic shock categories. Protein C levels improved significantly in recovery (p = 0.001) irrespective of immune status.

**Conclusion:**

Protein C levels were significantly lower in immunocompromised patients when compared to immunocompetent patients in severe sepsis and sepsis categories. Our study suggests a plausible role for APC in severely septic immunocompromised patients which need further elucidation.

## Introduction

The role of Protein C pathway in regulating thrombosis, fibrinolysis and inflammatory cascade in a septic patient is well established. Both baseline serum protein C and early reduction in protein C concentrations have been found to be independent predictors of outcome in severe sepsis [1,2]. This in combination with blunted generation of activated Protein C (APC) act in concert with reduced expression of thrombomodulin to contribute to a procoagulant state during sepsis [3,4]. The resulting intravascular deposition of fibrin and microvascular thrombosis contributes to the organ dysfunction and mortality in sepsis [5]. A phase III randomised controlled trial of activated protein C (APC) supplementation in severe sepsis (PROWESS), based on understanding of the pathophysiology, demonstrated a mortality benefit of APC therapy in severe sepsis that was statistically significant in the subgroup with protein C deficiency (p = 0.009) compared to those without protein C deficiency (p = 0.06) [6]. These findings were supported by the ENHANCE trial [7], although not by the ADDRESS trial [8]. Of note, in all these major trials, patients with immunocompromised states were excluded. One of the main reasons for exclusion of immunocompromised patients was their perceived high risk of bleeding. However, the advent of newer non-anticoagulant recombinant activated protein C molecule has allayed some concerns about the risk of bleeding which is a major drawback of APC as an effective anti-inflammatory drug [9,10]. Anecdotal evidence suggests that there may be a potential benefit of adding APC to standard therapy in septic shock after hematopoietic stem cell transplantation [11]. Preliminary data (Mesters et al) indicated that plasma protein C concentration diminished rapidly in the neutropenic patients who developed septic shock [12]. Thus the question still remains unresolved whether APC may have a role in the management of neutropenic sepsis. More fundamentally, it is not clear whether protein C and APC concentrations are reduced in immunocompromised sepsis. This study was therefore designed to assess and compare protein C levels in immunocompromised patients to immunocompetent patients during sepsis, severe sepsis, septic shock and recovery.

## Research design and the methods

This was a single centre prospective cohort study involving immunocompromised and immunocompetent patients in the haematology ward and the intensive care unit of Princess Alexandra Hospital, Brisbane. The study was approved by the Princess Alexandra Hospital human research ethics committee and informed consent was obtained either from patients or their next of kin. All patients admitted with a diagnosis of sepsis or those who developed sepsis during their admission were eligible. Three groups of patients were enrolled for the study: Immunocompetent patients with sepsis, immunocompromised patients with sepsis and immunocompromised patients without sepsis.

The diagnosis of SIRS, sepsis, severe sepsis and septic shock were based on standard criteria [13]. Patients were identified as *recovered *for the purpose of study when they no longer satisfied the sepsis criteria for at least 48 hours.

Patients were classified as *immunocompromised *if they were diagnosed with neutropenia (absolute count < 0.5 × 10^9^/L); myeloproliferative or lymphoproliferative condition; hyposplenism (based on history and imaging) or undergoing chemotherapy.

The *exclusion *criteria were:

a) Presence of hepatic failure (as protein C is secreted in hepatic cells)

b) History of spontaneous venous thrombosis and/or pulmonary embolism

c) Patients on Warfarin (Protein C is a vitamin K dependent protein)

d) Disseminated Intravascular Coagulation (DIC)

e) Patients already on activated protein C infusion

f) Failure to obtain consent

Patients were excluded from the study if they had DIC on basis of the modified overt diagnostic criteria of ISTH (International society of thrombosis and hemostasis) subcommittee on DIC. [14] In absence of any specific DIC criteria particularly for patients with hematopoietic malignancies and those on chemotherapy [15], we modified platelet count criteria for myelosuppressed patients to score 1 if reduction in platelet count by > 30% in last 24 hours not accompanied by similar fall in leucocyte count and score 2 if reduction in platelet count > 50% platelet in last 24 hours not accompanied by similar fall in leucocyte count.

The care of the patients was as per standard practice. No patient received APC infusion during the study.

### Protein C test methodology

Blood for protein C assay was collected in citrated tubes, double centrifuged before separation of plasma which was then aliquoted; snap frozen, labelled and stored at -75°C for assay. Fluorescence Immunoassay (*Triage *Protein C Meter, *Biosite*^†^) was used for rapid, quantitative determination of protein C in thawed aliquoted samples. The mean protein C concentration in healthy men and women, when tested using the protein C meter, is reported to be 4.23 μg/ml (median- 4.70 μg/ml) [16]. The coefficient of variation of the assay is reported to be 2-4% [17]. To ensure the accuracy, the supplied quality control device was run prior to each run of patient's samples. The test performance was also monitored using external protein C controls. Five samples (7% of total samples) were randomly tested twice and their results confirmed the validity of this commercial assay.

### Data collection and Blood sampling

The demographics, APACHE II [18] and SOFA scores [19] were collected on all patients. Post recruitment, patient's daily observation charts were closely followed up to assess if they satisfy criteria for sepsis, severe sepsis, septic shock or recovery. If patient's clinical condition fits in any of the abovementioned categories, then serum aliquots (for protein C assay) were prepared from their morning blood samples, if collected that day within last 6 hours, or else protein C test was requested to be done not later than by next morning. The blood samples were thus collected within 18 hours of clinical and biochemical observations that were noted for the purpose of calculating APACHE II score and SOFA score on all occasions. None of the patient had repeated sampling/measurements for any individual septic categories.

### Statistical Analysis

All statistical calculations were performed using the *StatsDirect *statistical software package. All continuous variables were analysed using basic descriptive statistics and univariate analysis was performed to obtain mean, standard deviation and/or confidence limits. The variables between two independent groups (e.g. immunocompromised and immunocompetent) were compared using unpaired t-test and the data was expressed as mean difference with 95% confidence interval (CI). A p < 0.05 was accepted as statistically significant. When the blood samples and other variables in the immunocompromised group obtained during different septic categories were compared against each other, one-way ANOVA for multiple comparison using Tukey-Kramer method (as unequal group sizes) was used.

## Results

Thirty eight patients were found to be eligible over a period of four months. Two patients were from non-English background and were excluded. Five patients refused consent. Thirty one patients participated in the study (22 Immunocompromised and 9 Immunocompetent).

a) Immunocompetent patients with sepsis (n = 9)

b) Immunocompromised patients with sepsis (n = 16)

c) Immunocompromised patients without sepsis (n = 6)

Table 1 shows the demographic data and the aetiologies of sepsis in all three groups. The groups were well matched in terms of age and the length of hospital stay. The causes for low immunity in the immunocompromised group were neutropenia (16/22), lymphoproliferative condition (4/22), hyposplenism (1/22) and minimally myelosuppressive chemotherapy (1/22). Five patients included in the neutropenia group were on myelosuppressive chemotherapy.

**Table 1:** Demographics in both groups of patients

	**Immunocompromised**		**Immunocompetent**
	**Non-Sepsis**	**Sepsis**	
n	6	16	9
Age in years (Range)	59 (29-74)	63.5 (45-81)	54 (22-72)
Females %	66%	25%	44%
Hospital stay (SD*)	17.3 days (15.4)	21.7 days (16.1)	24.9 days (18.9)
**Source of Sepsis**			
Pneumonia (CAP^§^)		2	3
Nosocomial Pneumonia		1	1
Abdominal sepsis		-	1
Urosepsis		-	1
Soft tissue infection		1	2
Bacteremia^Ж^		5	1
FUO (Fever of unknown origin)^Φ^		7	-
**Micro-organisms**			
Gram positives		Staph aureus (2)#	Staph aureus (1)#
		Staph hemolyticus (3)	Staph epidermidis (1)
Gram negatives		E.coli (1)	E. coli (2)
		Pseudomonas (1)	Pseudomonas (1)
		Klebsiella (1)	Legionella (1)
		Enterobacter cloacae (1)	Citrobacter koseri (1)
Fungus		Candida albicans (1)	Candida albicans (1)

* Standard Deviation, ^§ ^Community acquired pneumonia

^Ж ^Positive blood cultures some of which were related to catheter-related blood stream infections.

^Φ^FUO was considered when patients had persistent fevers despite intensive evaluation and diagnostic testing for more than a week as an inpatient in the hospital.

# (n) Number of patients in whom the specified micro-organism was incriminated as a cause of sepsis.

### Breakdown of Septic episodes

Among the immunocompromised septic patients (n = 16), 7 patients developed sepsis, 11 patients developed severe sepsis and 2 progressed to develop septic shock. The corresponding figures for the immunocompetent septic group (n = 9 patients) were 8, 6 and 3 respectively. None of the patient was represented more than once in any of the individual septic category.

There were two deaths among study patients, one of which was in the immunocompromised severe sepsis category and other one was in the immunocompromised septic shock category.

### Protein C concentration

Table 2 provides comparative data in the immunocompromised cohort measured during non-sepsis, sepsis, severe sepsis, septic shock and recovery. There was a statistically significant reduction in protein C concentrations in sepsis- 2.59 (95% *CI: *1.98- 3.21) μg/ml [p = 0.03], severe sepsis- 2.27 (95% *CI*: 1.63- 2.9) μg/ml [p = 0.001] and septic shock - 1.23 μg/ml, when compared to values during recovery - 3.82 (95% *CI: *3.23- 4.40) μg/ml. Moreover, there was no statistically significant difference between the protein C concentrations during recovery and non-sepsis - 3.96 (95% CI: 2.51- 5.41) μg/ml. Protein C levels, APACHE II scores and SOFA scores were similar across non-sepsis and recovery categories. Although, fibrinogen levels were elevated in sepsis, severe sepsis and septic shock, these did not reach statistical significance.

**Table 2:** Observations during sepsis, severe sepsis and septic shock in the immunocompromised cohort as compared to observations during recovery

**Variables**	**Immuno-compromised 'Non Sepsis'**	**Immuno-compromised 'Sepsis'**	**Immuno-compromised 'Severe sepsis'**	**Immuno-compromised 'Septic shock'**	**Immuno-compromised 'Recovery'**
**'n'**	6	7	11	2	13

**Age (Range)**	59 (29-74)	62 (45-81)	64 (57- 74)	64	59.5 (33- 81)

Protein C level in μg/ml (CL*)	3.96 (2.51- 5.41)	2.59 (1.98-3.21) p^§^- 0.031	2.27 (1.63- 2.9) p^§^- 0.0011	1.23	3.82 (3.23- 4.40)

SOFA score (CL*)	2.3 (0.6- 4.1)	2.9 (0.9- 4.7) p^§^- 0.94	4.6 (3.5- 5.6) p^§^- 0.024	12.5	2.4 (1.4- 3.4)

APACHE II score (CL*)	13.2 (9.8- 16.6)	14.6 (13.5- 15.6) p^§^- 0.233	17.6 (15.7- 19.6) p^§^- 0.0004	28	11.8 (9.8- 13.8)

Fibrinogen level in g/l (CL*)	2.94 (2.15- 3.73)	5.41 (3.34- 7.49) p^§^- 0.68	5.54 (3.63- 7.45) p^§^- 0.51	8.65	4.26 (3.53- 4.98)

**WBC (× 10^9^/l)**	1.16	4.94	0.5	0.9	4.7

**Platelets (× 10^9^/l)**	61.8	88.3	21.5	77.5	83

* Confidence limits (in brackets)

^§ ^p- p Value obtained when variables during different septic categories were compared to observations during recovery. There was no statistical difference among the variables (Protein C, APACHE II & SOFA scores) measured during non-sepsis and recovery.

p values for the septic shock cohort have not been provided because of the small sample numbers.

### Comparison of immunocompromised sepsis vs immunocompetent sepsis

Serum protein C concentrations were found to be significantly lower in the immunocompromised group compared to the immunocompetent group, particularly observed in severe sepsis [2.27 (95% *CI: *1.63-2.9) vs 4.19 (95% *CI: *2.87-5.52) μg/ml] (p = 0.01) and sepsis [2.59 (95% *CI: *1.98-3.21) vs 3.64 (95% *CI: *2.83-4.45) μg/ml] (p = 0.03). The results are summarised in Table 3.

**Table 3:** Observations in the immunocompromised and the immunocompetent cohorts during Sepsis, Severe Sepsis and Septic Shock

**Variables**	**Sepsis**		**Severe Sepsis**		**Septic Shock**	
	
	**Immuno-compromised**	**Immuno-competent**	**Immuno-compromised**	**Immuno-competent**	**Immuno-compromised**	**Immuno-competent**
**'n'§**	7	8	11	6	2	3

**Age (Range)**	62 (45-81)	56 (22-72)	64 (57-74)	53 (22-72)	64	65

**Protein C level in μg/ml (CL^Ж^)**	2.59* (1.98- 3.21)	3.64* (2.83- 4.45)	2.27** (1.63- 2.9)	4.19** (2.87- 5.52)	1.23	2.79

**SOFA score** **(CL^Ж^)**	2.9 (0.9- 4.7)	2 (0.7- 3.3)	4.6 (3.5- 5.6)	4.3 (2.4- 6.3)	12.5	8.3

**APACHE II score (CL^Ж^)**	14.6*** (13.5- 15.6)	8.5*** (4.8- 12.2)	17.6 (15.7- 19.6)	14.3 (10.3- 18.4)	28	22

**Fibrinogen level in g/l (CL^Ж^)**	5.41 (3.34- 7.49)	5.9 (2.19- 9.61)	5.54 (3.63- 7.45)	5.13 (2.01- 8.24)	8.65	7.9

**WBC (× 10^9^/l)**	4.94	13.8	0.5	17.6	0.9	31.2

**Platelets (× 10^9^/l)**	88.3	412.4	21.5	267.5	77.5	243.3

**PT^Φ^**	12^δ^	10.9^δ^	13.1	13.2	16	16

**Bilirubin^ξ^** **(in μmol/l)**	18.9^π^	11.5^π^	18.4^ω^	17.8^ω^	31.5	29

* **p = 0.03**, 95% CI (Mean Difference) = -1.97 to -0.1 and ** **p = 0.01**, 95% CI (Mean Difference) = -3.28 to -0.57, P value for difference in the Protein C levels between immunocompromised group and immunocompetent group during sepsis and severe sepsis respectively.

*** p = 0.006, P value for difference in APACHE II scores between immunocompromised group and immunocompetent group, when compared during 'sepsis'.

PT ^Φ ^- Mean Prothrombin time (seconds). Normal reference range for the laboratory is 9-12 seconds.

^δ ^p = 0.1, P value for difference in mean prothrombin time between two groups during sepsis.

Bilirubin ^ξ ^- Normal reference range for the laboratory is 5-17 μmol/l. ^π ^p = 0.1; ^ω ^p = 0.9; P value for difference in mean bilirubin levels between two groups during sepsis and severe sepsis.

p values for the septic shock cohort have not been provided because of the small sample numbers.

**'n'§**- Number of patients in each of the septic category. (Patients could be in more than one category depending on whether they developed sepsis, severe sepsis or septic shock. Hence, total number of septic episodes is more than total number of patients.)

**CL**^Ж ^- Confidence limits

### Sickness severity

SOFA scores were similar in both the groups across sepsis, severe sepsis and septic shock categories. Of note, the statistically significant difference in APACHE II scores between the immunocompromised and the immunocompetent groups should be looked at in light of the fact that all immunocompromised patients gained 5 extra points on chronic health adjustment (on account of being immunosuppressed) while calculating APACHE II scores and thus it does not imply any significant difference in the level of sickness among the two groups.

## Discussion

The principal finding of this study was that the plasma protein C concentrations in the immunocompromised septic patients were lower than those seen in the immunocompetent septic patients and the protein C levels improved with recovery. To our knowledge, this study represents the first clinical trial investigating protein C levels in immunocompromised patients as compared to immunocompetent patients across different septic categories.

### Mechanism of Protein C reduction in Sepsis

Protein C is an important component of the natural anticoagulant pathway. Under resting physiological conditions, protein C is continuously activated to maintain an anticoagulant milieu [20]. Severe sepsis is known to cause generalized endothelial dysfunction resulting in a pro-coagulant state in the microvasculature [21,22]. The activated coagulation cascade results in thrombin formation which then binds to thrombomodulin (TM). TM bound thrombin cause proteolysis of protein C and convert it to activated protein C (APC) which then down-regulates thrombin formation in negative feedback loop [23]. This activation of protein C is augmented by 20 fold in the presence of endothelial protein C receptor (EPCR) [24] which is known to have increased plasma concentrations in septic patients [25]. This interaction is a critical step in the host defence against sepsis since inhibition of protein C binding to EPCR (in baboon model) is shown to convert the response to sublethal concentrations of E.coli into a lethal response [26]. Protein C is thus consumed during development of severe sepsis thereby contributing further to the development of microvascular thrombosis and disseminated intravascular coagulation. Moreover, the cytokine response in sepsis may also result in decreased expression levels of TM and EPCR on endothelium and thus decreased activation of protein C [27]. These mechanisms may explain the reduction in plasma protein C concentrations in severe sepsis observed in our study.

However, our data indicates that plasma protein C levels are even lower in immunocompromised septic patients compared to the immunocompetent septic patients. There are various plausible mechanisms. A link between the protein C anticoagulant pathway and neutrophil functions is suggested by the observation that the soluble EPCR binds to activated neutrophils via Proteinase-3 and this binding is supported by β_2 _integrin involved in neutrophil signalling and cell-cell adhesion events [28]. It is possible that these molecular interactions are significantly affected by haematological malignancies, neutropenia or endothelial dysfunction induced by cytotoxic drugs thereby further impairing the protein C system in our immunocompromised septic patients. Most of our patients in the immunocompromised cohort had received chemotherapy at some stage for their underlying haematological condition. Chemotherapy has been shown to induce endothelial dysfunction that was observed in young adult survivors treated for acute lymphoblastic leukaemia in childhood [29,30] Malignancies and chemotherapy induced endothelial dysfunction might have predisposed our immunocompromised septic patients to have higher incidence of non-overt DIC resulting in increased consumption of protein C. It is also possible that protein C synthesis failed to keep up during the critical illness particularly when patients were on chemotherapy. Chemotherapy for breast cancer is known to decrease protein C concentrations [31]. Doxorubicin has been shown to induce dose and time dependent decrease in cell surface EPCR levels and increase in cell surface thrombomodulin in human umbilical vein endothelial cells (HUVEC) with a net effect of impaired capacity of HUVECs to convert protein C to activated protein C [32]. Since, activation of protein C system largely depends on TM and EPCR, it is important to study TM expression and EPCR expression in immunocompromised patients during non-sepsis state and in face of septic challenge in future studies. Other mechanisms that should be investigated are protein C consumption primarily due to DIC or reduced synthesis due to liver dysfunction. We excluded patients with overt DIC in this study and there were no significant differences between the two groups with respect to prothrombin time and bilirubin concentrations during any of the septic categories (Table 3).

### Possible Clinical significance of these results

The significance of protein C arises from published literature on activated protein C. Whilst we did not measure APC in this study, it is well recognised that circulating levels of APC strongly correlate (r = 0.75, p < 0.0001) with levels of protein C antigen in both healthy men and individuals with protein C deficiency suggesting that the circulating protein C concentration is the limiting factor in the rate of activation of protein C [33]. Therefore, protein C levels may reliably reflect APC levels in vivo.

### Study Limitations

This was a single centre study with small sample size. APC and other coagulation factors were not measured along with protein C. Moreover, the possible mechanisms of reduction in protein C such as soluble EPCR -neutrophils interaction in the immunocompromised patients, non-overt DIC scores and TM concentrations were not investigated and lastly, the relationship between protein C concentrations and outcome could not be examined owing to the small sample size.

## Conclusion

This preliminary study reveals significantly lower protein C levels in the immunocompromised patients during sepsis and severe sepsis as compared to the immunocompetent patients. There was a significant improvement in protein C levels with recovery in both groups of patients. Further studies are required to confirm the findings of our study in a larger setting, and investigate relationship between protein C concentrations and outcome in immunocompromised septic patients. Our pilot study might provide the platform for a future clinical trial designed to study benefits of activated protein C therapy in the immunocompromised septic patients.

## Key Messages

• Plasma protein C concentrations in immunocompromised patients are lower than immunocompetent patients, particularly observed in sepsis and severe sepsis.

• Protein C levels improved significantly with recovery irrespective of immune status.

• The study suggests a plausible role for APC in severely septic immunocompromised patients which need further elucidation in a randomized controlled clinical trial.

## Competing interests

The authors declare that they have no competing interests.

## Authors' contributions

RP, BV and PK were responsible for study design and data management. RP recruited patients, carried out Protein C assays and recorded all clinical data. RP and BV did statistical analysis and drafted the manuscript. BV, PK, RB, LN and DG were responsible for patient management. GD was responsible for managing protein C meter, preparing and preserving aliquots. RP, BV and PK did the data interpretation. BV, PK, RB and LN participated together with RP in editing the manuscript. All authors read and approved the final manuscript.
